# Molecular epidemiology of hepatitis C infection in Cyprus within the general population and high-risk cohorts

**DOI:** 10.1186/1756-0500-4-468

**Published:** 2011-10-31

**Authors:** Victoria L Demetriou, Leondios G Kostrikis

**Affiliations:** 1 Department of Biological Sciences, University of Cyprus, 75 Kallipoleos Avenue, P. O. Box 20537, 1678, Nicosia, Cyprus

## Abstract

**Background:**

Initial data on the molecular epidemiology of HCV infection in Cyprus showed a highly polyphyletic infection and multiple points of introduction into the general population. The continuation and expansion of this investigation is presented here including high risk groups.

**Findings:**

The samples include additional subjects from the general population, a group of inmates and HIV/HCV coinfected individuals, whose strains were amplified by RT-PCR and sequenced in partial Core-E1 and NS5B regions. The results confirm the broad genotype distribution and polyphyletic infection on the island, and no new subtypes were found. Monophyletic clusters between strains of the prisoners and the injecting drug users imply sharing of infected equipment, and highlight the risk of widespread transmission in these cohorts, although no spill-over to the general population was observed.

**Conclusions:**

The results of this study underline the impact of population movements and high-risk population groups on the changing molecular epidemiology of HCV, with strains moving to Europe from Asia, Africa and Eastern Europe by means of immigration and modern transmission routes.

## Background

Hepatitis C virus (HCV) infection is a major cause of chronic liver disease, cirrhosis and death, giving rise to a major global public health issue [1]. It is a highly genetically diverse RNA virus within the family *Flaviviridae*, and is separated into 6 confirmed genotypes and multiple subtypes that exhibit 30-35% and 20-25% nucleotide variation, respectively, at the level of complete genomes [2]. Determination of genotype is important due to clinical implications, but knowledge of the sequence diversity is also significant for studies on molecular epidemiology and evolution, and for classification and nomenclature [2,3]. Genotypes 1-3 display a worldwide distribution, while genotypes 4-6 are more geographically restricted and represent long-term endemic infection [4]. Subtypes 1b and genotype 2 are associated mainly with blood transfusions, especially in older patients, and subtypes 1a and 3a predominate among injecting drug users [5,6]. Genotype 4 is found mainly in North Africa and Mediterranean countries, Egypt in particular, but has recently been spreading to Europe and North America largely via intravenous drug users and immigrants [7], genotype 5 is restricted primarily to South Africa [8], and genotype 6 is found in Southeast Asia [9]. Globalisation is continually changing the face of worldwide HCV epidemiology as a result of modern transmission and human migration [10]. In the eastern Mediterranean region where Cyprus lies, HCV genotypes are not distributed uniformly [11-13]. Cyprus is a small island, but sits at a crossroad connecting Europe, Africa, and Asia, giving rise to a high rate of influx of tourists and political and economical immigrants from Eastern Europe and countries of the former Soviet Union, Africa and Southeast Asia. Also, as it is now a member state of the European Union, entry into Cyprus is easy, facilitating the introduction of new infectious diseases. The HCV genotype distribution and molecular epidemiology among the general population and intravenous drug users in Cyprus has been reported, revealing a polyphyletic infection with high genetic heterogeneity, small monophyletic clusters among the intravenous drug users, and the discovery of unclassified isolates [14,15]. The study is extended here by investigating additional samples from Cyprus, and specifically from high-risk cohorts, including incarcerated individuals and patients coinfected with HIV-1.

### Study subjects

Since the last study of the molecular epidemiology of HCV infection among the general population of Cyprus [14], sampling continued from 2008 to 2009 and blood samples were obtained from five (2 male) chronically infected HCV patients between the ages of 27 and 34, attending private clinics and public hospitals in Cyprus. All patients had been tested positive for HCV antibodies by a second-generation immunoassay (INNO-LiPA), and for HCV RNA by diagnostic RT-PCR (COBAS Amplicor, Roche Diagnostics, Branchburg, NJ, USA). The epidemiological features of the study subjects varied, as one patient was Cypriot and the rest were from Greece, Bulgaria and Belarus. Two of the participants stated that they had practised intravenous drug use, and for three the route of transmission is unknown.

Having previously obtained results from injecting drug users [15], and the fact that serving a prison sentence has been shown to be a significant risk factor for HCV infection, a cohort from the incarcerated population were included in this study as a high-risk cohort. A total of 25 samples from individuals serving a prison sentence had been taken for standard diagnostic purposes between June 2009 and January 2010 and any samples tested PCR-positive for HCV were retrospectively added to this study after written consent from the study subjects (16 individuals). Epidemiological and risk-behaviour details were collected from seven individuals. The participants were predominantly male (6, 85%), aged between 22-41 years, four (57%) were Cypriot, and three (43%) were of other nationalities (Greek, Georgian and Portuguese). All individuals who had been previously tested for coinfection with HBV and HIV (6, 85%) stated that they were tested negative for either infection. Three subjects had served multiple prison sentences. All interviewed persons stated that they had practiced intravenous drug use, six of which in Cyprus, and three had also practiced injecting drug use abroad. The median stated duration of injecting drug use was 5 years (interquartile range, 2-6 years). Regarding other possible routes of transmission and high-risk behaviour, none of the study subjects had ever had a blood transfusion, six (85%) had tattoos, all had practised unsafe heterosexual contact, and one participant believed that he had been infected with HCV during a fight within the correctional facilities.

Finally, a third group included in this study were HIV/HCV coinfected subjects, who are generally considered to arise from two types of risk groups: injecting drug users and men who have sex with men (MSM) [16-20]. From the 251 samples collected from HIV-1 seropositive patients from 2005-2009 for the study of the molecular epidemiology of HIV-1 in Cyprus [21], 14 were known to have HCV coinfection, according to results of routine diagnostic tests. All coinfected samples were subsequently included in this study, six of which (43%) were on antiretroviral therapy at the time of sampling. This cohort consisted of 10 male subjects (71%), with a median age of 34 years (interquartile range 32-45). The subjects displayed a variety of nationalities, with five (36%) being from Cyprus, four (29%) from Georgia, and one patient (7%) each from Bulgaria, Latvia, Italy, Britain and the USA. The stated routes of HIV-1 transmission for these patients was heterosexual contact for nine (64%), homosexual contact for two (MSM) (14%), intravenous drug use practises for two (14%), and one (7%) was a haemophiliac. No further specific details, however, were available regarding HCV transmission. All patients appeared epidemiologically unlinked, apart from one male and one female subject from Georgia, who were partners at the time of sampling [21]. Bioethics approval for the collection of, and experimentation on these samples was obtained from the Cyprus National Bioethics Committee (ΕΕΒΚ/ΕΠ/2005/03 and ΕΕΒΚ/ΕΠ/2009/02).

### Sampling and processing

All participants gave their informed consent, and clinical and epidemiological data were collected by use of anonymous coded questionnaires. Blood was collected from each participant by qualified personnel and plasma was isolated as previously described [14,22]. Viral RNA was extracted from 200 μl plasma using the QIAmp^® ^UltraSens^® ^Virus kit (Qiagen, Venlo, the Netherlands) following the manufacturer's instructions. All samples included in this study were investigated with a RT nested PCR in partial Core-E1 and NS5B regions of the HCV genome and all positive samples were sequenced as described previously [14].

### Molecular epidemiology within the general population and high-risk groups

**Figure 1:**
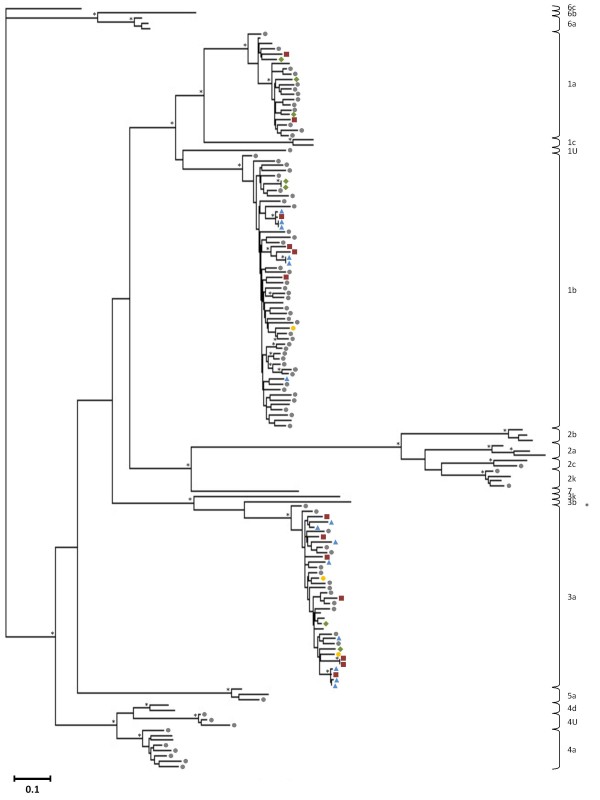
**Maximum likelihood tree of partial Core-E1 sequences**. Phylogenetic ML tree showing the genotype distribution of the HCV samples discovered in Cyprus sequenced in the Core-E1 genomic regions. HCV subtypes are designated with brackets on the right of each tree. U denotes unclassified strains. The samples are represented with different symbols for each cohort: grey circles denote samples from the general population from 2005-2008, yellow circles the general population from 2008-2010, red squares the incarcerated population, blue triangles the intravenous drug users, and green rhombus the HIV/HCV coinfected individuals. The general population 2005-2008 and the intravenous drug users have been previously described [14,15], and the rest are described in this study. Asterisks at branch nodes indicate bootstrap values of 70 and above. The genetic distance between any two sequences is denoted by the scale at the lower left side of each tree.

**Figure 2:**
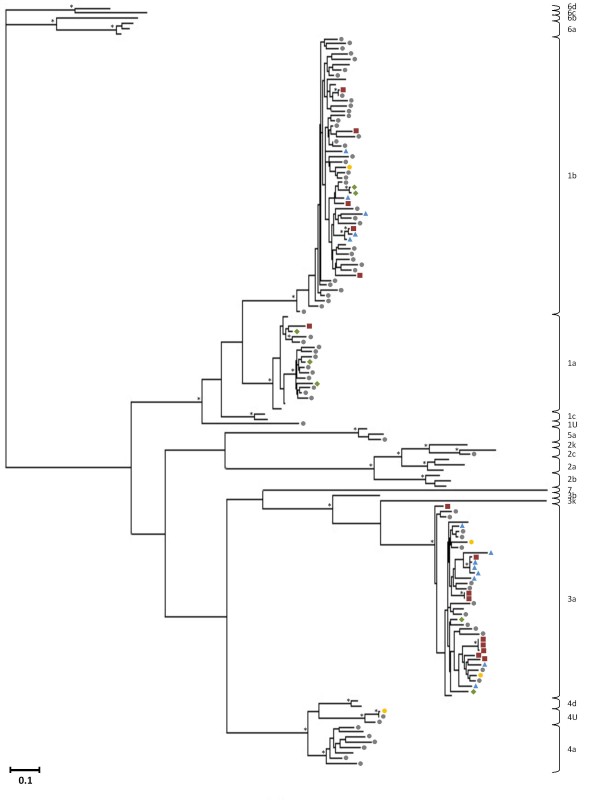
**Maximum likelihood tree of partial NS5B sequences**. Phylogenetic ML tree showing the genotype distribution of the HCV samples discovered in Cyprus sequenced in the NS5B genomic regions. HCV subtypes are designated with brackets on the right of each tree. U denotes unclassified strains. The samples are represented with different symbols for each cohort: grey circles denote samples from the general population from 2005-2008, yellow circles the general population from 2008-2010, red squares the incarcerated population, blue triangles the intravenous drug users, and green rhombus the HIV/HCV coinfected individuals. The general population 2005-2008 and the intravenous drug users have been previously described [14,15], and the rest are described in this study. Asterisks at branch nodes indicate bootstrap values of 70 and above. The genetic distance between any two sequences is denoted by the scale at the lower left side of each tree.

Phylogenetic analyses were performed on the Core-E1 and NS5B sequences derived from the general population, the incarcerated group and the HIV/HCV coinfected cohort, following multiple sequence alignments in MEGA v4 [23] with previously investigated HCV sequences from the general population and intravenous drug users in Cyprus [14,15] and reference strains of known subtypes derived from the Los Alamos HCV database [24] to investigate genotype distribution and sequence variability. Each set of aligned sequences was analysed with the model testing tool in MEGA v5 [25] and the best available evolutionary distance estimation model was chosen to construct Maximum Likelihood trees using Geneious v5.4 [26]. For the Core-E1 dataset the HKY approach was used with a gamma parameter of 0.45 and for the NS5B dataset the Kimura-2-parameter approach was used with a gamma parameter of 0.77 and a proportion of invariable sites (I) of 0.29. Bootstrap analysis was carried out with 500 replicates and values above 70 were considered sufficient for subtype assignment. Clusters were determined by an arbitrary threshold of genetic distance < 5%. The phylogenetic trees constructed from the sequences of the Core-E1 and NS5B regions are seen in Figures 1 and 2, respectively, where the samples from the various cohorts are represented with different symbols. HCV genotype 1 was the most frequent (47.7% in the Core-E1 region), followed by genotypes 3, 4, 2, and 5 (12.1%, 8.4%, 2.8%, and 0.9%, respectively in the Core-E1 region).

From the five new samples of the general population, four were PCR positive and they were classified as 3a (two strains, one from an injecting drug user), 1b (one strain) and unclassified genotype 4 (one strain). The latter was from a Bulgarian female and appears to belong to the unclassified group of genotype 4 isolated previously identified [14,27] and is the only strain from this group that clusters closely to another sample from Cyprus, but was only PCR positive in one of the two genomic regions. Hence, the genotypes found in this group are ones known to be present on the island [14,15].

From 25 samples taken for diagnostic testing from individuals serving a prison sentence at the state prisons, 13 were PCR-positive in the Core-E1 region and 16 in the NS5B region. The HCV genotypes found in this cohort were 3a (56%), 1b (31%) and 1a (13%) which have been associated with intravenous drug use [6,28,29], and showing a majority agreement with the genotype distribution in the Cypriot intravenous drug user population [15]. Phylogenetic analysis of the sequences obtained from the group of prisoners confirmed the polyphyletic nature of the local epidemic, but certain small monophyletic clusters also appeared among the injecting drug users and the inmates, suggesting a history of equipment sharing within small groups of users on the island, and that these inmates have had directly or indirect contact with the intravenous drug users discussed previously [15]. Within subtype 3a, sample HCVP05, from a 23 year old Cypriot male, clusters within an existing cluster of strains from Cypriot intravenous drug users (two male, one female) in both trees. The subjects of this cluster are also of similar age (19-24 years). Within the same subtype, two separate clusters are seen between strains of the prisoners' cohort. One is formed by samples HCVP04, HCVP07 and HCVP09, derived from one female and two male subjects, aged 21-35 years, two Cypriot and one of unknown nationality. This cluster is only observed in the NS5B tree, as two of the samples were PCR-negative in the Core-E1 region. The other small cluster is formed in both trees by samples HCVP15 and HCVP16, from two male subjects ages 39 and 49, one Cypriot and one of unknown nationality. Within subtype 1b, sample HCVP17, from a 42 year old Cypriot male, groups closely with an existing cluster of strains from Cypriot and Greek male injecting drug users in the trees of both genomic regions. It is interesting that the Cypriot subjects who cluster together here are in a similar age group (39-47 years) that indicates the probability of a long duration of drug use. In the same subtype, another small cluster is observed, involving sample HCVP12, from a 24 year old Georgian male, and a strain of the general population, CYHCV93, from a 39 year old Georgian male who was infected through intravenous drug use and, interestingly is a 2 k/1b recombinant strain, known to circulate in certain injecting drug user circles in Eastern Europe [30]. However, sample HCVP12 was PCR negative in the Core-E1 region, and so it cannot be seen if it was 2 k in this region and whether it would cluster with the same sample from the general population. This phylogenetic relationship in the NS5B region and the similar demographic and epidemiological characteristics (Georgian nationality, male, 25 years old, history of intravenous drug use), could mean that the strain in question is also a recombinant of this type, but this would have to be confirmed with additional sequence data. The small clusters are formed between strains from individuals with common characteristics (nationality, age group, injecting drug use), demonstrating that such users are more likely to associate with each other, increasing the danger of sharing injecting drug use equipment. These results underline the significance of rapid and uncontrolled transmission between injecting drug users and inmates. Even though intravenous drug use is generally considered to be the main risk factor for prisoners, the routes of transmission could be multiple, due to the range of risk behaviours in prisons, including unsanitary tattoo and piercing procedures and getting into fights [31-33], which were all stated behaviours from the study subjects of this cohort. No strains from the general population were included in these clusters, illustrating that the HCV epidemic in the high-risk groups runs in parallel to the HCV-infected general population.

From the samples from individuals coinfected with HIV and HCV, eight were PCR positive for both regions, and sequences could be obtained from seven samples. The results showed the existence of HCV genotypes 1a (3 samples), 1b (2 samples) and 3a (2 samples), which are all genotypes previously found among coinfected subjects, but in different geographical locations [17,19,34-37]. Two strains cluster closely together (genetic distance < 1%) in subtype 1b in both trees, and they are derived from two individuals who were a heterosexual couple at the time of sampling, strongly implying transmission from one individual to the other, but it cannot be certain whether infection was through sexual practises or household transmission. Their HIV-1 strains also display a similar clustering both in the full genome analysis [21] and in investigations of the gag, pol (Pr/RT) and env regions separately [22]. No other strains from this cohort form clusters and none show significantly close phylogenetic relationships with samples from neither the general population nor the other high-risk cohorts. It is likely that the routes of transmission for the two viruses were the same in many cases. Of all the stated risk behaviours in this group, the most improbable transmission route for HCV is the heterosexual contact, where it may have been acquired through another unknown route. Regarding the country of origin, the Cypriot study subjects in this cohort carried HCV genotypes 3a and 1a, Georgians carried 3a and 1b, and both the Latvian and Bulgarian subjects were infected with HCV subtype 1a. Concerning risk behaviours, the MSM carried HCV genotypes 3a and 1a. The subjects who were infected with HIV through heterosexual contacts were infected with all three HCV subtypes found in this cohort, possibly signifying the fact that they may have transmitted HCV through different routes, or simply representing the fact that they were more individuals in this subgroup than for other HIV transmission routes in this cohort. The intravenous drug user whose HCV strain was sequenced was infected with 1a, a strain typically found in injecting drug users.

All the high-risk groups (intravenous drug users, incarcerated population and HIV/HCV coinfected individuals) display a similar genotype distribution, due to common risk behaviours. HIV/HCV coinfection has been associated mainly with injecting drug use and MSM behaviour, and even among coinfected MSM, injecting drug use is significantly associated, as well as multiple other risk behaviours [17-19,34-39]. The small number of HCV-positive samples found in the HIV-infected cohort from 2005-2009 could imply a low prevalence of HCV infection among MSM on the island at the moment. However, as this risk group is getting increasing attention with regards to HCV transmission in Europe, this prevalence could also change in Cyprus in the near future. Even though an increasing incidence of genotype 4 has been reported in Europe, in intravenous drug users and MSM coinfected with HIV [13,19,36,39-42], no such cases were found in the high-risk groups, even though they were present in the general population. More studies need to be carried out to establish a more representative global picture of the epidemiology of HCV in individuals coinfected with HIV.

The overall data obtained here show a polyphyletic infection, certain monophyletic clusters among injecting drug users and prisoners of similar age and nationality, and no spill-over to the general population. The general picture of the molecular epidemiology of HCV infection obtained from an epidemiologically diverse set of samples from various cohorts confirms a high genetic heterogeneity, broad genotype distribution, and multiple points of introduction, similar to the pattern seen for HIV in Cyprus [22]. These data are representative of the geographical position of Cyprus, which lies at a crossroad between three continents resulting in population movements that appear to greatly influence the epidemiology of HCV on the island. Also, the diverse background of the study subjects emphasise the impact of immigration from various countries and increasing occurrence of injecting drug abuse on the island's HCV epidemic, highlighting the risk of widespread transmission in this high-risk cohort. The results here support that the point of entry of HCV on the island or in any of the cohorts investigated was not due to a single-transmission introduction.

In conclusion, the results of this study reveal the molecular epidemiology of the HCV infection in Cyprus in more detail and demonstrate the changing HCV epidemiology, with genetically diverse strains finding their way into Europe from the Middle East, Africa and Eastern Europe, by means of injecting drug abuse and immigration. Also, the broad genotype distribution and multiple points of infection have implications on clinical management, and highlight the urgent need for risk reduction and prevention strategies.

### Nucleotide Sequence Accession Numbers

The GenBank accession numbers of the reference strains used in the construction of phylogenetic trees of the partial Core-E1 and NS5B sequences are 1a, AF511950, EF407419 and NC_004102; 1b, AY587016, D11355 and EF032892; 1c, AY051292 and D14853; 2a, AB047639, AY746460 and D00944; 2b, AB030907, AF238486 and D10988; 2c, D50409; 2k, AB031663; 2k/1b, AY587845; 3a, AF046866, D17763 and X76918; 3b, D49374; 3k, D63821; 4a, DQ418788 and Y11604; 4d, DQ418786 and DQ516083; 5a, AF064490 and Y13184; 6a, AY859526, DQ480513 and Y12083; 6b, D84262; 6c, EF424629; 7a, EF108306. The accession numbers for the previously described strains found in the general population and intravenous drug users in Cyprus are: EU684661-EU684737 and GQ332540-GQ332553 for the Core-E1 region and EU684591-EU684660 and GQ332554-GQ332565 for the NS5B region.

The accession numbers for sequences obtained in this study and submitted to GenBank are HQ537010-2 and HQ537033-6 for the general population sequences, HQ537013-25 and HQ537037-52 for the sequences of the incarcerated population, and HQ537026-32 and HQ537053-9 for the sequences of the HIV/HCV coinfected group.

## Competing interests

The authors declare that they have no competing interests.

## Authors' contributions

VLD assisted in coordination and design of the study, carried out the sample preparation and extraction, the molecular experimental procedures, the sequence alignments and phylogenetic analysis, and drafted the manuscript. LGK conceived, coordinated and designed the study, participated in data interpretation and analysis, and assisted in drafting the manuscript. All authors have read and approved the final manuscript.
